# Burnout and metabolic syndrome among healthcare workers: Is subclinical hypothyroidism a mediator?

**DOI:** 10.1002/1348-9585.12252

**Published:** 2021-07-19

**Authors:** Meng‐Ting Tsou, Jau‐Yuan Chen

**Affiliations:** ^1^ Department of Family Medicine MacKay Memorial Hospital Taipei City Taiwan; ^2^ Department of Occupation Medicine MacKay Memorial Hospital Taipei City Taiwan; ^3^ Department of MacKay Junior College of Medicine, Nursing, and Management New Taipei City Taiwan; ^4^ Department of Family Medicine Chang‐Gung Memorial Hospital Linkou Branch Taiwan; ^5^ Chang Gung University College of Medicine Taoyuan Taiwan

**Keywords:** burnout, healthcare workers, metabolic syndrome, Taiwan

## Abstract

**Objectives:**

Evidence suggests that subclinical hypothyroidism (SCH) is associated with burnout and metabolic syndrome (MetS). We examined the relationship between burnout and MetS among healthcare workers (HCWs) and investigated the potential mediation of SCH.

**Methods:**

This cross‐sectional study included HCWs from a tertiary medical center; demographic data were obtained using a questionnaire. Burnout was evaluated according to the Chinese version of the Maslach Burnout Inventory–Health Services Survey (MBI–HSS). MetS and thyroid function data were obtained from a physical check‐up. Logistic regression models were used to evaluate the adjusted odds ratio (aOR), and mediation analysis was employed to examine the mediation effect.

**Results:**

Among 945 non‐doctor/nurse and 1868 doctor/nurse staff, MetS was 30% and 14%, respectively, and the prevalence of burnout was nearly 6.5%. The results showed that burnout induced higher aOR of MetS in the doctor/nurse group (1.27, 95% confidence interval [CI]: 1.05‐3.62). Thyroid‐stimulating hormone (TSH) showed a positive association factor of MetS in doctor/nurse group‐adjusted burnout (aOR = 1.15, 95% CI: 1.01‐4.19). A higher TSH level was associated with an increased odds of MetS in younger doctor/nurse staff with burnout syndrome (aOR = 1.74; 95% CI: 1.04‐3.22). There was a borderline significant mediation effect of SCH in the association between burnout and MetS in doctor/nurse staff.

**Conclusions:**

The results showed that higher TSH levels were positively associated with burnout and MetS in doctor/nurse professionals, especially in the young cohort. Burnout may rely on the borderline mediation effect of SCH, which is likely to affect MetS.

## 1 INTRODUCTION

Healthcare workers (HCWs) are required to respond to patients and families, making their work stressful,^1^ especially nurses and physicians who need to take care of patients and undergo repetitive and continuing exposure to the ill, dying, and death.^1^ Nurses and physicians have similar work characteristics, including working in shifts and excessive working hours.^1^, ^2^ HCWs have a higher risk of adverse health effects, including metabolic syndrome (MetS) and burnout.^2^, ^3^, ^4^, ^5^ Studies in hospitals in Taiwan observed burnout in nurses (66%), physician assistants (61.8%), physicians (38.6%), administrative staff (36.1%), and medical technicians (31.9%).^3^ The prevalence of MetS for the entire staff was 12.0%, physicians (18.3%), and nurses (6.6%).^4^

Studies that evaluated the association between burnout and MetS among the nursing staff working in a primary healthcare clinics in Brazil and a tertiary hospital in Mexico showed that burnout syndrome increases the risk of MetS by 1.45 [95% confidence interval (CI): 1.17‐1.81]^6^ as well as abdominal circumference with the domains of burnout (emotional exhaustion) [adjusted odds ratio (aOR) = 14.95; 95% CI: 1.5‐148.7].^7^ The results of our recent study conducted in a tertiary hospital in Taiwan showed that burnout was responsible for a 1.70‐fold higher risk of MetS among nursing staff (95% CI: 1.04‐3.05).^8^ However, few studies have explored the association between burnout and MetS among HCWs, including other hospital staff.

A recent meta‐analysis suggested that subclinical hypothyroidism (SCH) was positively associated with the prevalence of MetS, especially among Asian population.^9^ In another 4‐year prospective cohort study, participants with MetS were associated with a significantly increased risk of developing SCH compared to those without MetS at the baseline.^10^ Because SCH increases cholesterol, blood pressure, and visceral fat levels, understanding why it is associated with MetS is not difficult.^11^

Acute stress has been shown to cause the transient activation of the hypothalamic–pituitary–thyroid (HPT) axis, whereas prolonged stress is associated with decreased activity.^12^ Asberg et al^13^ found significant differences in triiodothyronine (T3) and thyroid‐stimulating hormone (TSH) levels among employees from sick leave, burnout, and healthy groups, as an early and sensitive biochemical predictor of chronic stress. However, Guo et al^14^ found no significant association between burnout symptoms and TSH, T3, and thyroxine (T4), which is consistent with the existing literature on both burnout and biochemical markers.^14^, ^15^ Chronic stress has direct effects on the HPT–adrenal axis and the sympathetic nervous system, which results in the development of visceral obesity, type 2 diabetes, atherosclerosis, and MetS.^16^

**FIGURE 1:**
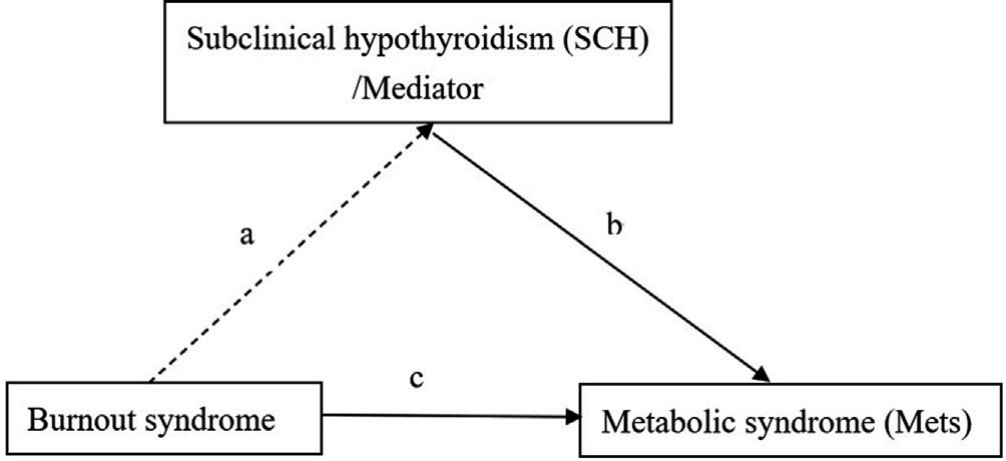
Diagram of medication effect model for subclinical hypothyroidism showing the link between burnout and metabolic syndrome. a*b, causal mediation effect. There is a causal mediation effect if the range of average causal medication effect size doesn't include “0”

Studies have reported that burnout is positively associated with MetS in nursing staff.^6^, ^7^, ^8^ Moreover, evidence has shown that SCH is associated with burnout and MetS (edges a and b in Figure 1). The TSH level is reportedly higher in chronic stress conditions, suggesting that it induces changes in the psycho‐endocrine system. Therefore, this study intends to use retrospective cohort data to explore the relationship between burnout and MetS in HCWs (including doctor/nurse and non‐doctor/nurse groups). The most considerable hypothesis is to use the mediation analysis model to understand whether SCH plays a mediator role leading to burnout to induce MetS (edge c in Figure 1).

## 2 MATERIALS AND METHODS

### 2.1 Study design and participants

**FIGURE 2:**
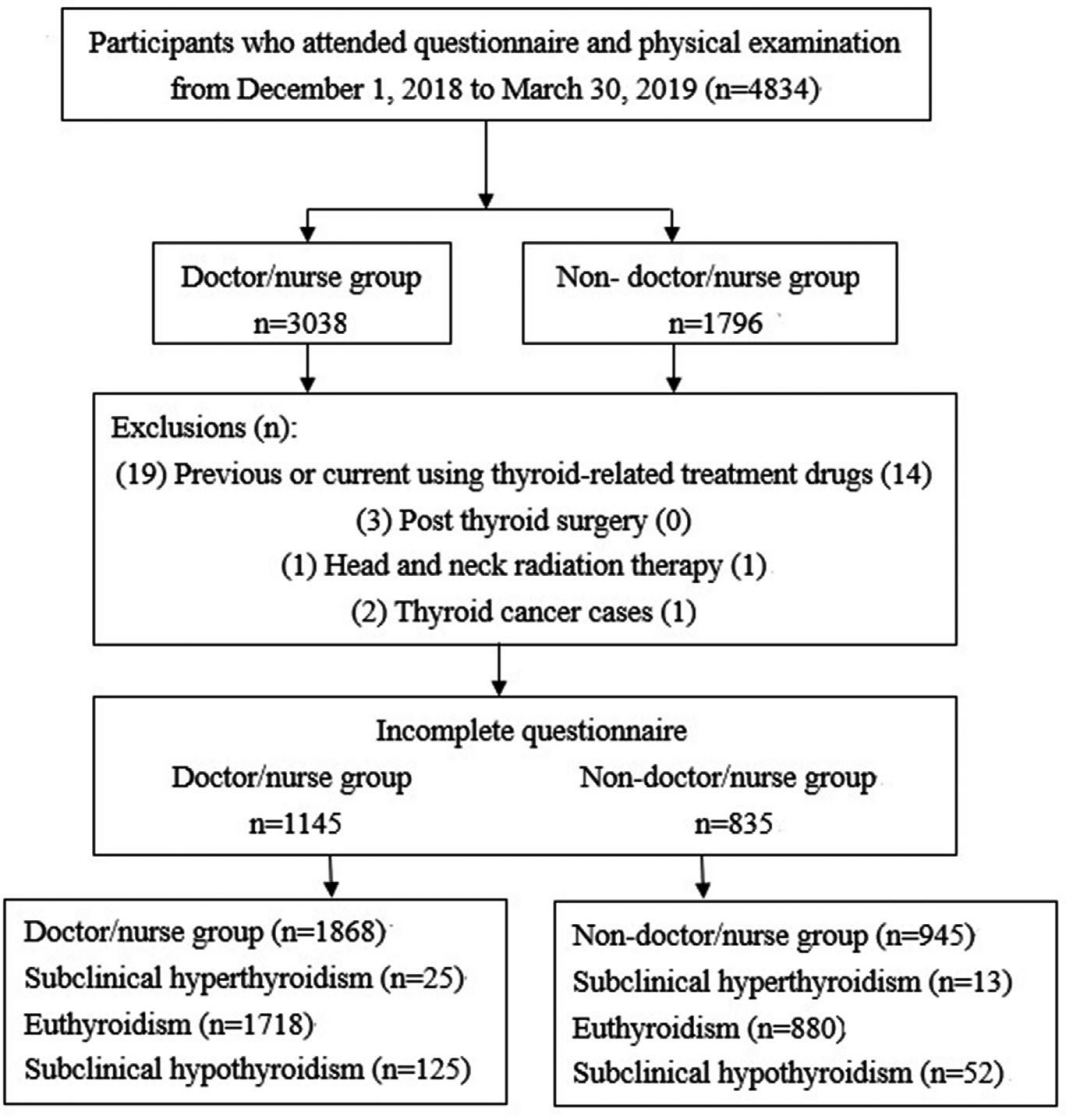
Study design flowchart

This cross‐sectional study was conducted at the Health Evaluation Center in Mackay Memorial Hospital, a 2000‐bed tertiary teaching center in both the Taipei/New Taipei, Taiwan branch. This region has an estimated population of 2.67 million. Questionnaires were administered between December 2018 and March 2019. Based on the findings of previous studies,^1^, ^2^, ^3^, ^4^, ^5^ different departments of HCWs have different work styles, prevalence of MetS and burnout. For these reasons, we divided HCWs into two groups (doctor/nurse and non‐doctor/nurse). Non‐doctors/nurses included technicians, administrative staff, pharmacists, radiologists, nutritionists, and others. A total of 3038 doctor/nurse and 1796 non‐doctor/nurse participants were invited after excluding the procedure (see Figure 2), and 1868 and 945 cases from the two groups completed the questionnaires. The actual response rates were 62.1% (1868/3013) in doctor/nurse group and 53.1% (945/1780) in non‐doctor/nurse group.

#### 2.1.1 Sociodemographic data and working characteristics

Information was obtained using structured questionnaires, which were designed according to the Institute of Labor, Occupational Safety, Ministry of Labor (Survey of Perceptions of Safety and Health in the Work Environment in 2016 Taiwan; ILOSH105‐M309)^17^; the following data were collected: socioeconomic status, working characteristics, job burnout syndrome status, and mental health. Analysis of the internal consistency reliability of each part of the scale showed the Cronbach's *α* to be between 0.82 and 0.93, indicating good reliability.

#### 2.1.2 Anthropometric measurements

Baseline characteristics and anthropometric measurements, including age, body height, body weight, waist circumference (WC), and blood pressure (BP), were collected and recorded by trained nurses blinded to the patients’ information at Health Evaluation Center.

#### 2.1.3 Laboratory data acquisition and analysis

Sample collection and analysis were performed in a standard laboratory with international accreditation (ISO‐15189). All participants were requested to fast for more than 8 hours before venous blood sampling and were collected in a BD Vacutainer SSTTM (Becton Dickinson) sample collection tube. Sample collection and analysis principles were based on the standard requirements given in the Clinical Laboratory Standards Institute guidelines (specimen choice, collection, and handling; approved guideline H18‐A3).

A Hitachi 7170 Automatic Analyzer (Hitachi Corp.) was used to measure the levels of fasting glucose, lipid markers (including low‐ and high‐density lipoprotein cholesterol [HDL‐C; homogeneous enzymatic colorimetric assay]), and triglycerides (TGs). Free T4 (fT4) and TSH levels were determined by an immunoradiometric assay using a commercial kit (DiaSorin). Quality control and instrument operation were performed according to standard procedures dictated by the Clinical Laboratory Standards Institute guidelines. Samples were assessed in triplicates, and the final values (after quality control) were confirmed to be in the linear range using an internal standard.

#### 2.1.4 Burnout assessment

Burnout domains were evaluated using the Chinese version of the Maslach Burnout Inventory–Health Services Survey test (MBI–HSS) as per Lu et al^18^ (Cronbach's *α*, 0.84). Three domains of the MBI–HSS were evaluated: emotional exhaustion (EE), depersonalization (DP), and personal accomplishment (PA). The MBI–HSS includes 22 questions in total, and the frequency of occurrence is scored using a 7‐point Likert scale from 0 (never felt) to 6 (felt every day).^18^ EE scores γ 27, DP scores γ 13, and PA ϕ 31 were categorized as high job burnout.^18^ The EE and DP showed directly related to burnout, while, on the contrary, PA showed a negative association.^18^ The internal consistency coefficients (Cronbach's *α*) of the three dimensions of EE, DP, and PA are 0.77, 0.83, and 0.82, respectively.^18^

#### 2.1.5 Brief Symptom Rating Scale (BSRS‐5)

Mental health was assessed using the BSRS‐5. It is a self‐assessment questionnaire, which includes five items. It requires respondents to report whether they felt tensed, blue, irritated, or inferior or had any problems falling asleep in the past week. Responses are rated on a scale of 0‐4, with 0 being “nothing” and 4 being “extremely.” The total score ranges from 0 to 20.^19^ When a score of ≥6 was used as the cutoff point for psychiatric cases, the BSRS‐5 was divided into four groups: “no symptoms” (0‐5), “mild” (6‐9), “moderate” (10‐14), and “severe” (>15).^19^

The internal consistency was analyzed by Cronbach's *α* coefficient, the BSRS‐5 coefficients were from 0.77 to 0.90,^19^ and the test‐retest reliability coefficient was 0.82. Using a score of ≥6 as the cutoff point for psychiatric cases, the BSRS‐5 accurate classification rate was 76.3% (sensitivity: 78.9%; specificity: 74.3%; positive predictive value: 69.9%; negative predictive value: 82.3%).^19^

#### 2.1.6 Classification according to MetS and thyroid function

To analyze the metabolic score, the classification of the National Cholesterol Education Panel of the National Treatment Program for Adults III (NCEP‐ATP III) with specific cutoff points was used for the Taiwanese population for abdominal obesity.^20^

Individuals who met the MetS criteria with at least three of the following five components were defined as exhibiting MetS: (a) WC ≥90 cm for men and 85 cm for women; (b) HDL‐C < 40 mg/dL for men and <50 mg/dL for women; (c) TG levels ≥150 mg/dL; (d) BP ≥130/85 mmHg or treatment for hypertension (HTN); and (e) fasting blood glucose (SFB) ≥ 100 mg/dL or treatment for type 2 diabetes mellitus.^20^ The reference ranges for fT4 and TSH were 1.0‐1.71 ng/dL and 0.4‐4.0 mU/L, respectively.^21^

### 2.2 Ethics approval

The study protocol was evaluated and approved by the Human Research Ethics Committee of Mackay Memorial Hospital (project research number 18MMHISO150). All participants provided written informed consent. Data confidentiality was preserved, considering ethical issues, such as autonomy and respect for people, and all the guidelines of the Declaration of Helsinki were followed. To ensure data confidentiality, participant identification information was replaced with a folio number.

### 2.3 Statistical analysis

A descriptive analysis was performed to characterize the population sample. Data are presented as means ± standard deviations for continuous variables, and numbers and percentages are presented for categorical variables. According to the cutoff point of the NCEP‐ATP III, MetS factors were classified into two categories, and frequencies and percentages were calculated for each factor. For comparison between the groups, a Student's *t* test was used to analyze continuous variables, and the chi‐square test was used for categorical variables. Multivariate logistic regression analyses were performed to investigate the possible associations among MetS, burnout, age, sex, and biomarkers after adjusting for education level. In patients with and without burnout syndrome, the association of TSH level and MetS prevalence after age stratification was used for multivariate logistic regression in the doctor/nurse group. All analyses were performed using SPSS 22 (IBM Corp.) for Windows. Two‐sided *P* values of <.05 were considered statistically significant.

Based on the mediation model in Figure 1, R package “Mediation” (V.4.4.5) was used to analyze the mediation effect of TSH level (binary variable, low/normal, and high TSH level) in the relationship between burnout syndrome (binary variable, no and yes) and MetS (binary variable, no and yes) (R V.3.2.5 software).^22^ The nonparametric bootstrap method was used to estimate the average causal mediation effect (ACME) size by adjusting potential confounding factors. A high TSH level was identified as a mediator if the range of ACME did not include 0.

## 3 RESULTS

### 3.1 Characteristics of the participants

A total of 1868 doctor/nurse and 945 non‐doctor/nurse participants were included in this study. The mean age and women prevalence of the non‐doctor/nurse group were significantly higher than those of the doctor/nurse group (age, 44.68 ± 11.20 vs 35.29 ± 10.96 years; women: 81.58% vs 93.36%, *P* < .001; Table 1). The education level of the doctor/nurse group was significantly higher than that of the non‐doctor/nurse group.

**TABLE 1:** Characters and prevalence of MetS, MetS factors, thyroid function, TSH subgroups, mental character, and work character among hospital employees

Variables	Non‐doctor or nurse (n = 945)		Doctor and nurse (n = 1868)		*P* value
Age, y (mean, SD)	44.68 (11.20)		35.29 (10.96)		<.001
Women, n (%)	771	81.58	1744	93.36	<.001
Education level, n (%)					
≦Senior high school	326	34.50	45	2.41	<.001
College	499	52.80	1698	9.90	
≧Graduate school	120	12.70	125	6.69	
*Metabolic factors*					
WC, cm (mean, SD)	80.14 (10.85)		75.41 (10.47)		<.001
SBP, mmHg (mean, SD)	123.66 (19.33)		116.86 (14.56)		<.001
DBP, mmHg (mean, SD)	72.15 (12.21)		67.49 (10.21)		<.001
BS, mg/dL (mean, SD)	100.40 (22.19)		93.54 (15.72)		<.001
TG, mg/dL (mean, SD)	115.21 (45.85)		86.42 (34.67)		<.001
HDL‐C, mg/dl (mean, SD)	58.05 (15.44)		61.55 (14.22)		<.001
*Metabolic syndrome, n (%)*	283	29.95	261	13.97	<.001
Central obesity, n (%)^a^	370	39.15	513	27.46	<.001
Elevated blood pressure, n (%)^b^	352	37.25	395	21.15	<.001
Hyperglycemia, n (%)^c^	373	39.47	343	18.36	<.001
Hypertriglyceridemia, n (%)^d^	217	22.96	218	11.67	<.001
Low HDL‐C, n (%)^e^	243	25.71	322	17.24	<.001
*Biomarkers*					
Free T4, ng/dL (mean, SD)	1.22 (0.30)		1.18 (0.39)		.45
TSH, mU/L (mean, SD)	1.76 (1.16)		2.04 (1.62)		.037
TSH subgroup (n, %)					
<0.4 mU/L	13	1.38	25	1.34	.55
0.4‐4 mU/L	880	93.12	1718	91.97	.08
>4 mU/L	52	5.50	125	6.69	.02
*Mental characteristics*					
Burnout (n, %)	61 (6.46)		122 (6.53)		.94
Maslach burnout inventory (MBI) (mean, SD)					
Emotional exhaustion	21.76 (6.36)		26.99 (6.78)		<.001
Personal accomplishment	25.02 (5.30)		27.56 (5.32)		<.001
Depersonalization	8.84 (5.57)		11.41 (5.80)		<.001
Emotional exhaustion, ≧27 (n, %)	252	26.67	659	35.28	<.001
Personal accomplishment, ≦31 (n, %)	308	32.59	609	32.60	.86
Depersonalization, ≧13 (n, %)	389	41.12	462	24.73	<.001
BSRS‐5 score (mean, SD)	4.15 (3.33)		5.62 (3.91)		<.001
BSRS‐5, n (%)					
0‐5	706	74.71	1099	58.83	<.001
6‐9	162	17.14	456	24.41	
10‐14	68	7.20	252	13.49	
15‐20	9	0.95	61	3.27	
*Work characteristics*					
Seniority, n (%)					
<2 y	160	16.85	370	19.81	.001
2‐4 y	164	17.37	307	16.43	
4‐10 y	243	25.74	566	3.30	
>10 y	378	40.04	625	33.46	
Working hours/wk, n (%)					
≦45 h	741	78.39	1058	56.64	<.001
46‐50 h	185	19.60	609	32.60	
51‐59 h	14	1.48	115	6.16	
≧60 h	5	0.53	86	4.60	
Work style, n (%)					
Regular class	692	73.23	753	40.31	<.001
Night shift	17	1.80	116	6.21	
Three shifts	236	24.97	999	53.48	

Abbreviations: BP, blood pressure; BS, blood sugar; BSRS, Brief Symptom Rating Scale; HDL‐C, high density lipoprotein‐cholesterol; n, numbers; SD, standard deviation; TG, triglyceride; WC, waist circumference.

^a^ Waist circumference ≥90 cm in men or ≥80 cm in women.

^b^ SBP ≧ 130 mmHg or DBP ≧ 85 mmHg or self‐reported hypertension.

^c^ Fasting blood glucose ≥100 mg/dL or self‐reported diabetes mellitus.

^d^ TG ≥ 150 mg/dL.

^e^ HDL‐C < 40 mg/dL in men or <50 mg/dL in women.

### 3.2 MetS prevalence, MetS factors, thyroid function, and TSH subgroups stratified by different professions

Table 1 shows the prevalence and condition of MetS and burnout in these two groups. The results showed that the prevalence of MetS and MetS factors of non‐doctor/nurse staff was statistically significantly higher than that of doctor/nurse staff (MetS prevalence: total, 19.3%; non‐doctor/nurse vs doctor/nurse: 29.95% vs 13.97%, *P* < .001). In the non‐doctor/nurse group, hyperglycemia and central obesity ranked first and second, respectively. In the doctor/nurse group, central obesity and high BP ranked first and second, respectively.

The result of the thyroid function analysis showed that TSH was higher in the doctor/nurse group (2.04 ± 1.62 vs 1.76 ± 1.16, *P* = .037); fT4 showed no statistical difference. Further analysis by TSH level was divided into three groups: low TSH level (<0.4 mU/L); normal TSH level (0.4‐4 mU/L); high TSH level (>4 mU/L); the result showed the higher proportion of doctor/nurse staff in the high TSH level group (6.69% vs 5.50%, *P* = .02).

### 3.3 The prevalence of burnout, burnout domains, BSRS‐5, and work style stratified by different professions

Table 1 shows that no statistical difference was found in the prevalence of burnout between the two groups (burnout prevalence: total, 6.5%; non‐doctor/nurse vs doctor/nurse: 6.46% vs 6.53%, *P* = .94), but analysis of the three domains showed that the scores and prevalence of EE and DP were higher in the doctor/nurse group than in the non‐doctor/nurse group. The PA score was lower in the non‐doctor/nurse group, but the prevalence was higher. The BSRS‐5 score and the proportion of BSRS‐5 score ≧6 subgroup were found to be higher in the doctor/nurse group than in the non‐doctor/nurse group (5.62% vs 4.15%, *P* < .001; 41.17% vs 25.86%, *P* < .001).

The seniority of the doctor/nurse staff was primarily less than 10 years. Relatively, 40% of the non‐doctor/nurse staff were more than 10 years. The working hours of the doctor/nurse group were higher than those of the non‐doctor/nurse group. Approximately 42% of doctor/nurse staff reported working more than 46 hours per week; nearly 60% of the doctor/nurse group needed to perform three shifts and night shifts (*P* < .001).

### 3.4 aOR of burnout, age, sex, and thyroid function among HCWs varies according to profession with MetS

In Table 2, multivariate logistic regression analysis was performed to identify predictive factors (independent variables included burnout, age, sex, and thyroid function) of HCWs with MetS (dependent variable) after adjusting for educational level, BSRS‐5, and working style. Results showed that burnout demonstrated a higher aOR in the doctor/nurse group (1.27, 95% CI: 1.05‐3.63). Female gender demonstrated a higher aOR of inducing MetS for all HCWs. Higher TSH demonstrated a higher aOR of having MetS in the doctor/nurse group (1.15, 95% CI: 1.01‐4.19). Age and fT4 demonstrated no statistical significance between burnout and MetS, but a higher fT4 was found to demonstrate lower aOR of MetS (0.28, *P* = .068).

**TABLE 2:** Adjusted odds ratios (aORs) and 95% CI of the burnout, age, sex, and thyroid function among HCW varies according to profession with MetS

Variables	Non‐doctor or nurse			Doctor and nurse		
	aOR	95% CI	*P* value	aOR	95% CI	*P* value
Burnout (yes vs no)	0.97	0.221‐4.29	.97	1.27	1.05‐3.62	.01
Age (y)	1.04	0.98‐1.10	.19	0.97	0.90‐1.04	.41
Sex (women vs men)	3.33	1.07‐5.39	<.001	2.78	1.10‐3.78	.02
TSH	0.96	0.73‐1.27	.78	1.15	1.01‐4.19	.04
Free T4	0.93	0.71‐1.21	.57	0.28	0.07‐1.10	.068

Note

Adjusted by education level, BSRS‐5 and work characteristics.

Abbreviations: aOR, adjusted odds ratio; BSRS, Brief Symptom Rating Scale; CI, confidence interval; free T4, free thyroxine; TSH, thyroid‐stimulating hormone.

### 3.5 Analysis of MetS prevalence by stratified age, burnout, and TSH subgroups among HCWs varies according to profession

After stratifying for age and burnout status, the doctor/nurse group showed higher TSH levels and exhibited higher MetS prevalence than other subgroups in the younger burnout syndrome group (aORs: 1.74, 95% CI 1.04‐3.22) (Table 3a). No statistically significant difference was found in other subgroups (Table 3a).

**TABLE 3:** The association between TSH subgroup and prevalence of MetS by using logistic regression analysis stratified by age and burnout in (a) doctor/nurse group; (b) non‐doctor/nurse group

Subgroup	No. of MetS (%)	aOR (95% CI)	*P* value
**(a)**			
20‐40 y and burnout (n = 72)			
Normal TSH (49)	8 (16.3)	Ref	
Low TSH (7)	1 (14.3)	0.84 (0.45‐1.73)	.67
High TSH (16)	3 (18.8)	1.74 (1.04‐3.22)	.04
20‐40 y and no‐burnout (n = 1155)			
Normal TSH (1078)	85 (7.9)	Ref	
Low TSH (8)	0 (0.0)		
High TSH (69)	6 (8.7)	1.12 (0.62‐1.72)	.45
41‐73 y and burnout (n = 50)			
Normal TSH (41)	13 (31.7)	Ref	
Low TSH (2)	0 (0.0)		
High TSH (7)	2 (28.6)	0.86 (0.13‐9.83)	.63
41‐73 y and no‐burnout (n = 591)			
Normal TSH (550)	135 (24.5)	Ref	
Low TSH (8)	2 (25.0)	1.04 (0.15‐3.64)	.55
High TSH (33)	6 (18.2)	0.63 (0.11‐2.77)	.19
**(b)**			
Burnout (n = 61)			
Normal TSH (55)	11 (20.0)	Ref	
Low TSH (2)	0 (0)		
High TSH (4)	0 (0)		
No‐burnout (n = 884)			
Normal TSH (825)	256 (31.0)	Ref	
Low TSH (11)	3 (27.3)	0.74 (0.15‐1.97)	.19
High TSH (48)	13 (27.1)	0.72 (0.10‐1.88)	.14

Note

Normal TSH: TSH = 0.4‐4 mU/L; low TSH: TSH < 0.4 mU/L; high TSH > 4 mU/L.

Abbreviations: aOR, adjusted odds ratio; CI, confidence interval; MetS, metabolic syndrome.

In the non‐doctor/nurse group, the sample size was too small after age grouping. Table 3b shows the stratified analysis based on the level of TSH and burnout, and the results were not statistically significant.

### 3.6 Mediation analysis

Table 3 found a significant difference between TSH level, MetS, and burnout in the doctor/nurse group. Mediation analysis was conducted to explore the role of SCH in the link between burnout and MetS in the doctor/nurse group. The ACME of SCH for all staff was 0.0641 (95% CI: 0.1054‐0.3137, *P* = .19) after adjusting for confounding factors. ACMEs were 0.0578 (95% CI: 0.1014‐0.2824, *P* = .14) and 0.0804 (95% CI: 0.0010‐0.4289, *P* = .05) for non‐burnout staff and burnout staff, respectively (Table 4). A borderline statistical difference was noted among the burnout staff.

**TABLE 4:** Mediation analysis of SCH in the link between burnout and MetS in doctor/nurse group

	Effect size (95% CI)^a^	*P* value
Burnout		
ACME	0.0804 (−0.0010 to 0.4289)	.05
ADE	0.0206 (−0.1047 to 0.5129)	.71
Non‐burnout		
ACME	0.0578 (−0.1014 to 0.2824)	.14
ADE	0.0208 (−0.1053 to 0.5499)	.73
All staff		
ACME	0.0641 (−0.1054 to 0.3137)	.19
ADE	0.0212 (−0.1018 to 0.5864)	.73

Abbreviations: ACME, average causal mediation effect; ADE, average direct effect; BSRS, Brief Symptom Rating Scale; MetS, metabolic syndrome; SCH, subclinical hypothyroidism.

^a^ Adjusted for age, sex, education level, BSRS‐5 and working characteristics.

## 4 DISCUSSION

In our study, burnout and female gender induced a higher association with MetS in the doctor/nurse group. According to previous research conclusions, working character, age, and gender can influence the prevalence of MetS and burnout.^6^, ^7^, ^8^, ^11^ According to Schaufeli et al,^23^ patients/clients hope to have more concern, care, empathy, and compassion, and the character of job has a continuous and close relationship with patients/clients, the higher the risk of burnout, such as HCWs, police, and teachers. In our results, the working character of the doctor/nurse group echoes the conclusions mentioned in the previous studies.^23^

Our research found that the prevalence of MetS among the total HCWs was 19.3%, which was higher than that of other tertiary hospitals in Taiwan (12%).^3^ The prevalence of the non‐doctor/nurse and doctor/nurse groups in this study was 29.95% and 13.97%, respectively. The former was consistent with the general population in a previous study in Taiwan [25.5% of men and 31.5% of women had MetS].^24^ The prevalence of the latter is lower than that of the general population, but it is similar to the prevalence of the doctor/nurse group by Yeh et al (12.5%).^4^ The main reason may be that the staff in the doctor/nurse group are in the young female age group (compared to the non‐doctor/nurse group), which is consistent with the fact that the prevalence of MetS in women under the age of 50 is lower than that of men in the same age group in a previous study^25^ and the healthy worker effect.^3^

In our study, the prevalence of MetS in the doctor/nurse group (13.9%) was higher than that of Taiwanese workers in different jobs observed by Wei et al (12.1%),^26^ which means that even the doctor/nurse group with the health worker effect^3^ still has a high prevalence of MetS. According to previous studies, the main reason is the work environment, including work stress, long working time, and burnout, which make doctor/nurse staff at greater risk of being diagnosed with MetS.^27^

Previous studies proved that long hours of work (>10 h/d) and night shifts in the hospital were strongly associated with MetS, cardiovascular disease, and adiposity‐based chronic disease.^28^, ^29^, ^30^ One systematic review showed no conclusion between shifting work and chronic disease, which may be ascribed to the influence of confounding factors, including race, age, gender, occupational type, and chronic stress.^31^ Other mental factors from the workplace, such as depression and job stress can contribute to MetS in HCWs.^32^ Our previous study found similar results in the nurse department, but no other staff was included in this study.^8^ In this study, we found that burnout and females still demonstrated a higher aOR of MetS in doctor/nurse staff after adjusting for factors, but no association in the non‐doctor/nurse staff, which supported that burnout played the leading role in MetS.

In further stratified analysis, we found that the 20‐40‐year‐old burnout population with high TSH levels had higher aOR of MetS after adjusting for other confounding factors compared with other age groups and the non‐burnout group. The meta‐analysis by Gómez‐Urquiza et al^5^ showed that younger age was a significant factor in burnout.^5^ At the same time, our results found that the young doctor/nurse group with burnout was at a higher MetS aORs (particularly a high TSH level). However, similar results were not found in the non‐doctor/nurse group. Therefore, SCH may be an important factor in burnout and MetS among young doctor/nurse staff.

A previous study mentioned that work stress could lead to altered thyroid hormone levels causing hypothyroidism or SCH,^33^ contributing to insulin resistance and visceral fat accumulation. These two conditions may be caused by the role of the thyroid on lipid and glucose metabolism as well as BP regulation.^33^ A strong relationship between depression and SCH was discussed.^34^ A large retrospective cohort study in Korea by Kim et al^35^ showed that the TSH level as a continuous variable significantly predicted depressive symptoms in females and suggested that SCH is a major neuroendocrine condition predisposing females to developing depression symptoms.^35^ In our study, we found that depression was higher in the doctor/nurse group. According to the conclusions of previous studies,^35^ a higher proportion of females and depression in the doctor/nurse group was related to SCH.

In our study, 42% of the doctor/nurse staff worked for >46 hours per week, and nearly 60% of the doctor/nurse group was required to work three shifts and night shifts. Previous studies have shown that TSH is significantly higher in the night shift group than in the day shift group, suggesting that females are more sensitive to night shift work than males.^36^, ^37^ In our study, the proportion of females and shift work in the doctor/nurse group was higher than those in the non‐doctor/nurse group, and high TSH levels were found in the former group, which supports the previous conclusions.

According to previous studies, night shifts and depression are related to SCH,^33^, ^34^, ^35^, ^36^, ^37^ and SCH related to MetS and burnout.^23^, ^27^, ^33^ In the causal mediation analysis, our results showed that work stress had two different effects on MetS: a borderline effect mediated through SCH in the doctor/nurse group with burnout, but not in this group without burnout. Regarding the pathway between burnout and MetS, there is inconsistent evidence that biomarkers are activating the HPA axis, such as serum cortisol.^15^ Other factors, including working character and emotions, could be the direct influencing factors of MetS due to burnout, and these factors need to be further analyzed.

### 4.1 Limitations

The present study had some limitations. First, this was a single‐institution study. Second, causality in the associations cannot be analyzed because this was a cross‐sectional study. Third, the actual response rate was low (62.1% [1868/3013] in the doctor/nurse staff and 53.1% [945/1780] in the non‐doctor/nurse staff). Fourth, since the sample size of the collected participants among different departments is not large enough, we cannot perform a more detailed subgrouping analysis. In the future, it would be helpful to perform a large‐scale sample analysis to distinguish different departments of HCWs. Finally, several factors, such as the severity and duration of burnout and the influence of other neuroendocrine hormones, were not evaluated; these factors will be evaluated in a future study.

## 5 CONCLUSION

The present study showed a significant association between burnout and MetS in a young female and high TSH cohort in the doctor/nurse profession. Our previous study found that burnout involving seniority at work, night shifts, working hours, and depressive mood might lead to a higher risk of MetS.^7^ This preliminary study showed that SCH might play a mediating role between burnout and MetS. Hence, we advocate TSH screening and burnout monitoring for HCWs, particularly women and young doctor/nurse staff. Overall, the results of this study can be regarded as preliminary and need to be further investigated in a prospective cohort.

## DISCLOSURES

*Approval of the research protocol*: The study protocol was examined and approved by the Human Research Ethics Committee at Mackay Memorial Hospital (project research number 18MMHISO150). *Informed consent*: Informed written consent was obtained from each participant before starting the study. *Registry and the registration no. of the study*: N/A. *Animal studies*: N/A. *Conflict of interest*: The authors declare no conflict of interests.

## AUTHOR CONTRIBUTIONS

MT Tsou participated in the design and conception of the study and its coordination, acquisition of data, carried out statistical analysis, and drafted the manuscript. JY Chen participated in the conception of the study and participated in the design of the study and reviewed analysis and manuscript. All authors reviewed the manuscript and had read and agreed to the published version of the manuscript.

## Data Availability

No additional data are available.
